# Unusual Clinical Presentation of Nephrotic Syndrome With Significant Renal Vein Thrombosis in a Young Male: A Case Report and Literature Review

**DOI:** 10.7759/cureus.43705

**Published:** 2023-08-18

**Authors:** Bair Cadet, Kathryn Leonard, Jaswinder P Singh, Ishmam Alam

**Affiliations:** 1 Department of Internal Medicine, Nassau University Medical Center, East Meadow, USA; 2 Department of Internal Medicine, American University of the Caribbean, Cupecoy, SXM

**Keywords:** edema, proteinuria, primary membranous glomerulopathy, vein thrombosis, nephrotic syndrome

## Abstract

Primary membranous glomerulopathy is the most common cause of idiopathic nephrotic syndrome, with increasing recognition as an autoimmune-mediated disease. We present the case of a 31-year-old Hispanic male with no prior medical or family history, presenting with one month of dyspnea on exertion, lower extremity, and periorbital edema with a recent diagnosis of pulmonary embolism. Upon further imaging, renal vein thrombosis was discovered with significant lab dysfunction concerning nephrotic syndrome. Further, a workup of kidney biopsy and serum antibody levels revealed the cause to be anti-phospholipase A2 receptor (PLA2R)-mediated.

## Introduction

This article was previously presented as a meeting abstract at the 2022 ASN Annual Kidney Week Meeting on November 04, 2033.

Primary membranous glomerulopathy is the most common cause of idiopathic nephrotic syndrome, with increasing recognition as an autoimmune-mediated disease [1]. We present the case of a 31-year-old Hispanic male with no prior medical or family history, presenting with one month of dyspnea on exertion, lower extremity, and periorbital edema with a recent diagnosis of pulmonary embolism. Upon further imaging, renal vein thrombosis was discovered with significant lab dysfunction concerning nephrotic syndrome. Further, a workup of kidney biopsy and serum antibody levels revealed the cause to be anti-phospholipase A2 receptor (PLA2R)-mediated.

## Case presentation

A 31-year-old Hispanic male with no medical history presented with left-sided chest pain, shortness of breath on exertion, and swelling in his face and legs for the past day. The patient was recently diagnosed with pulmonary embolism one-month prior and was discharged on anticoagulation, which he had reported compliance with taking daily. He reported that the chest pain and dyspnea felt similar to his initial hospitalization one month ago, but the swelling was a new symptom. Upon examination, the patient had severe pitting edema to bilateral lower extremities up to the knee and had fullness to the periorbital region. On auscultation, the patient was tachycardic but had no abnormalities on the cardiovascular or pulmonary exam. He was hypoxic, saturating 94% on room air. His abdominal exam was also significant for distension with the presence of ascites.

**Figure 1:**
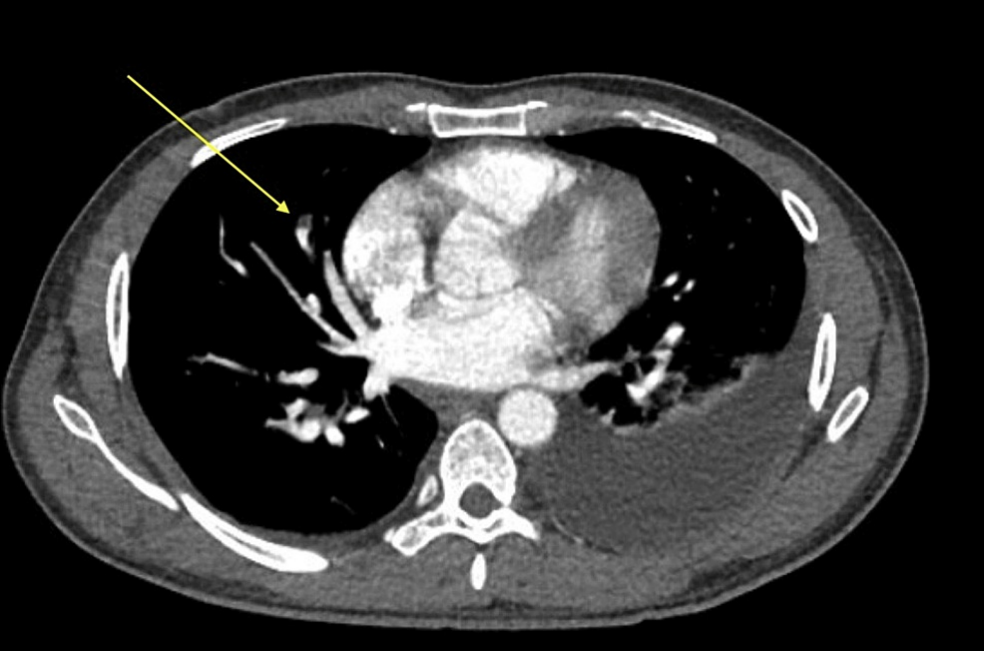
Subsegmental pulmonary embolism involving the anterior right middle lobe (yellow arrow)

**Figure 2:**
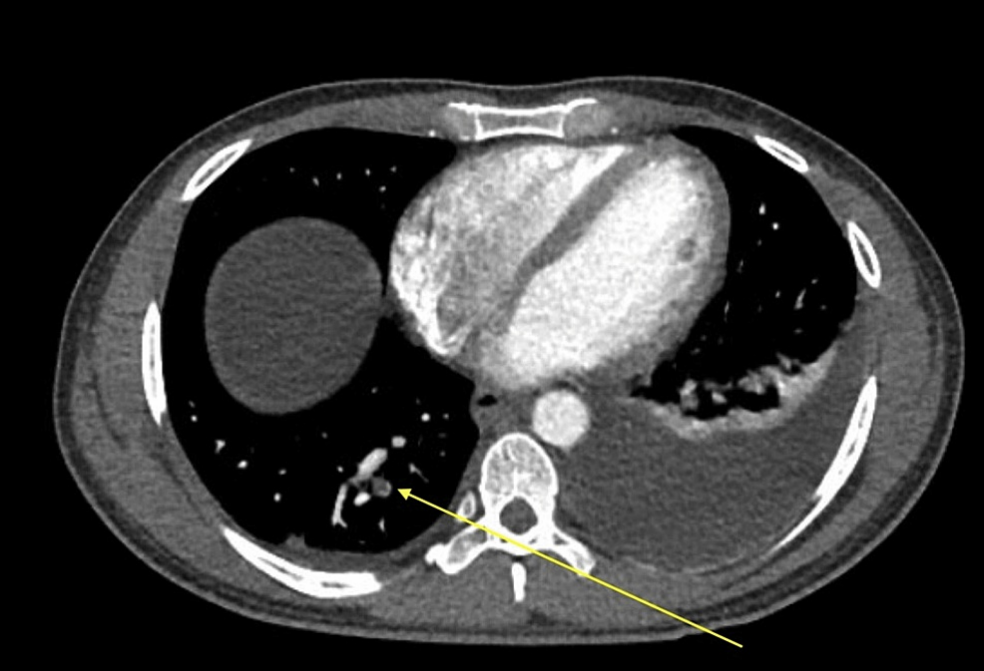
Subsegmental pulmonary embolism involving the posteromedial right lower lobe (yellow arrow)

**Figure 3:**
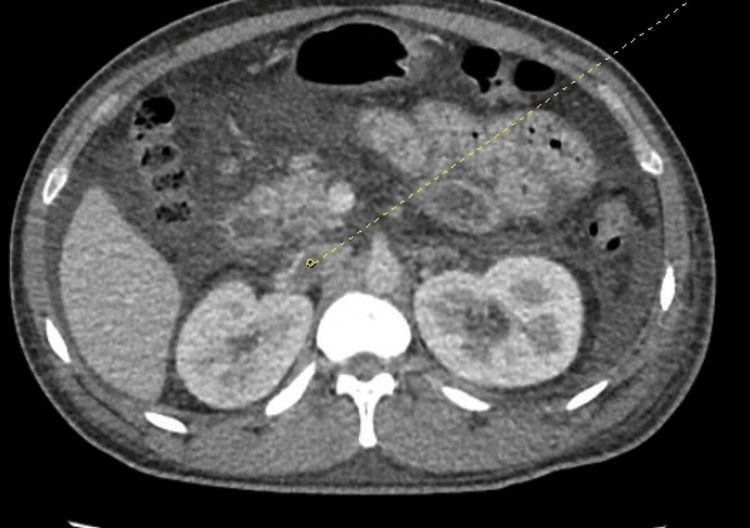
Right renal vein thrombus extending into the inferior vena cava (yellow dotted line)

**Figure 4:**
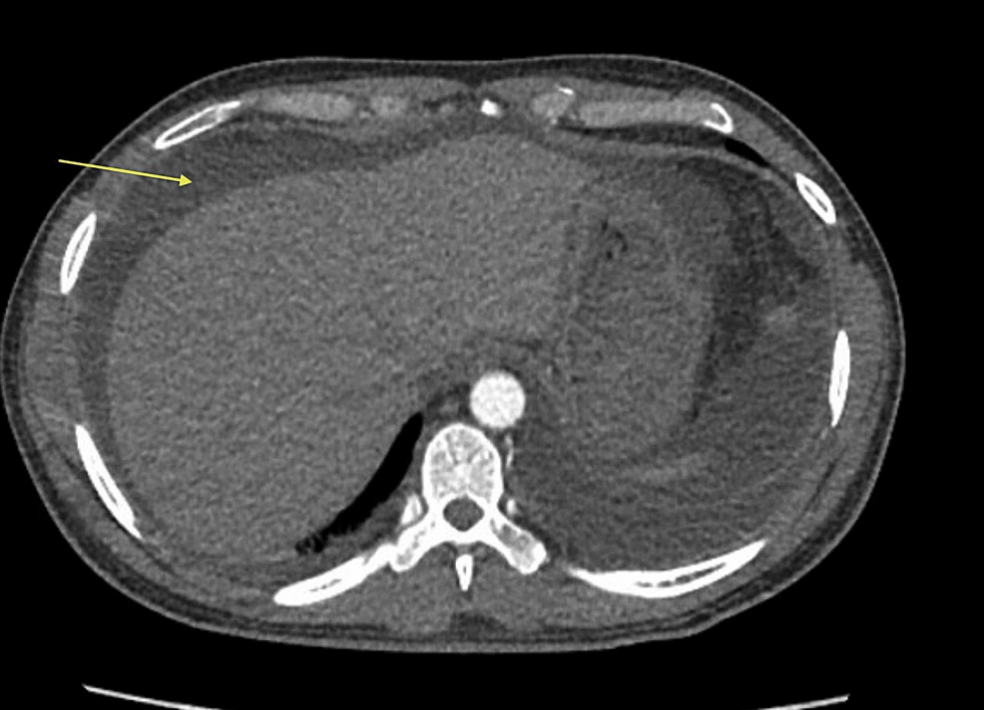
Ascites (yellow arrow)

Initial thorax and abdomen/pelvis imaging revealed subsegmental pulmonary emboli, right renal vein thrombus extending into the inferior vena cava, and significant volume ascites (Figures 1-4). Laboratory workup revealed severe hypoalbuminemia of less than 1.3 gm with a total protein of 3.4 g/dL. Urine studies, lipid panel, and thrombophilic workup, including antiphospholipase A2 receptor antibodies (anti-PLA2R), anticardiolipin antibodies, lupus anticoagulants, assays for protein c and s, and genetic testing for factor V mutation. were obtained. The lipid panel revealed elevated total cholesterol of 424 mg/dL and low-density lipoprotein of 339 mg/dL. Initial urinalysis showed significant proteinuria of >300 mg/dL, with additional urine creatinine and urine protein 24-hour studies both elevated with a ratio of 23.5 (Tables 1-2).

**Table 1:** Lipid panel HDL: high-density lipoprotein, LDL: low-density lipoprotein

Lipid panel	Patient labs value	Reference range
Total cholesterol	424 mg/dL	Less than 200 mg/dL
Triglycerides	119 mg/dL	Less than 150 mg/dL
HDL	59 mg/dL	Greater than 50 mg/dL
LDL	339 mg/dL	Less than 100 mg/dL

**Table 2:** Urine protein to creatinine ratio uPCR: urine protein to creatinine ratio

24-hour studies	Patient lab value	Normal range
Urine creatinine	906 mg/24 hr	500-2000 mg/d
Urine protein	21261 mg/24hr	Less than 80 mg
uPCR	23.46	Less than 0.2

**Figure 5:**
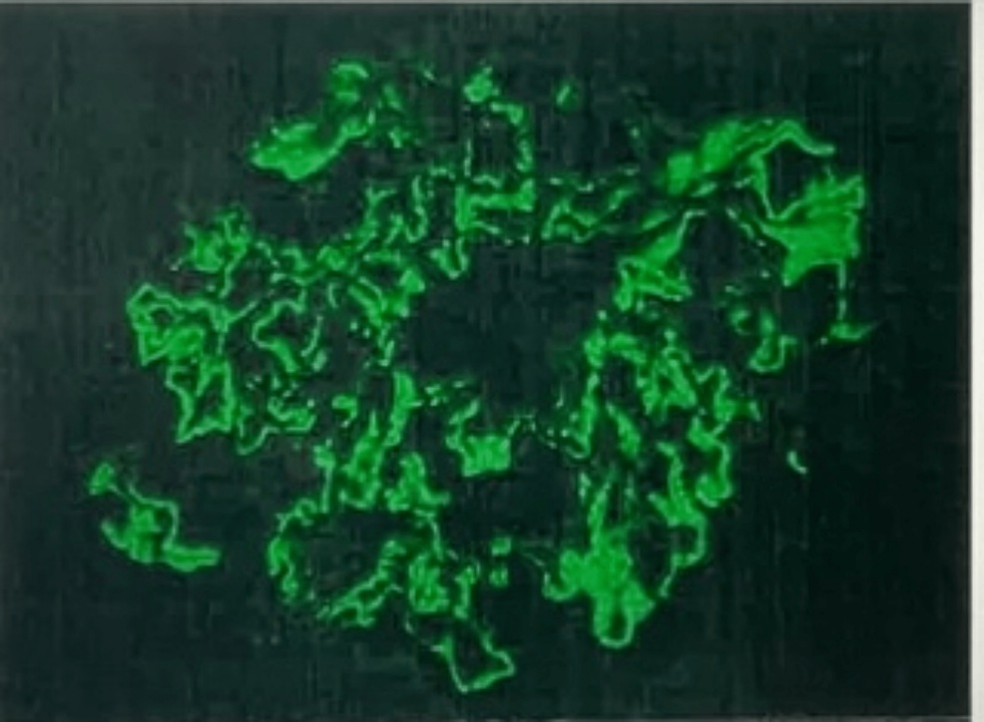
Immunofluorescent staining for IgG

The serum PLA2R antibody panel was also positive with a 47 RU/mL level. The remainder of the thrombophilic workup was unremarkable. Subsequent kidney biopsy was obtained and revealed immunofluorescence staining for IgG, C3, kappa, and lambda chains and phospholipase A2 receptor to be positive in the distribution of the glomerular subepithelial deposits, thus confirming primary (anti-PLA2R-mediated) membranous glomerulopathy (Figure 5).

## Discussion

The approximate incidence of membranous nephropathy (MN) is 8 to 10 cases per million in the adult population, with the most common demographic being Caucasians over 40 years old [1]. In contrast, our patient was a previously healthy 31-year-old Hispanic male who developed acute nephrotic syndrome secondary to MN with no known underlying familial history or other predisposing conditions.

The presentation of MN is classically associated with findings such as lower extremity edema, periorbital edema, gross proteinuria, and ascites. Uniquely, our patient reported an initial presentation of shortness of breath, chest pain, and palpitations. Preliminary evaluation and diagnosis were for unprovoked pulmonary embolism with anticoagulation treatment. However, our patient presented one month later with lower extremity edema, periorbital swelling, shortness of breath, and ascites. Clinical evaluation was significant for marked hypoalbuminemia, proteinuria, and pulmonary emboli with renal vein thrombosis. Although this patient had sought treatment previously for a similar presentation, imaging of the abdomen and pelvis at that time was deferred for unknown reasons.

This highlights the importance of including abdominal and renal vein imaging when there is clinical suspicion of nephrotic syndrome, as up to 35% of patients have renal vein thrombosis [2]. Whereas intrauterine renal vein thrombosis is most commonly due to factor V Leiden, most adult cases of renal vein thrombosis are associated with disruptions of Virchow’s triad (hypercoagulability, endothelial damage, and venous stasis). More specifically, patients with primary MN have a higher risk of developing venous thrombosis due to their hypercoagulable state. CT scan with contrast remains the imaging modality of choice for renal vein thrombosis and offers nearly 100% sensitivity and specificity. If a patient cannot tolerate contrast, magnetic resonance angiography may be considered at the cost of reduced sensitivity and specificity. Early anatomical changes in renal vein thrombosis, such as renal hyper-echogenicity and renal enlargement, may be seen with ultrasonography in about 90% of patients [3]. One particular cohort study including 430 in-hospital patients showed a 10.6% occurrence of venous thrombosis with a higher frequency of deep vein thrombosis than renal vein thrombosis [4]. Another significant finding in this large cohort study was that the levels of anti-PLA2R antibodies were found to be significantly higher in patients with VT compared to those without, even after adjusting for the influence of hypoalbuminemia and hypercholesterolemia as risk factors.

Antibodies to PLA2R have been reported in 70% of patients with primary MN with genetic links to HLA DQA1 and PLA2R1 genes [1-5], and significant associations have been found between anti-PLA2R antibody levels and long-term outcomes in patients. The lower titer is associated with a better rate of spontaneous remission and time to remission in those requiring therapy. Anti-PLA2R correlates with disease activity as well [6]. While further investigation is required, survival analysis of one study conducted among 90 prevalent patients with biopsy-proven MN revealed that high levels of PLA2R antibodies were linked with active disease and significantly linked to overall outcome with a higher likelihood of declining renal function [7]. The level of PLA2R antibodies is an important marker to trend as it can dictate the type of treatment and whether to initiate immunosuppressive therapies among patients. Additionally, changes in circulating PLA2R antibodies can occur more rapidly than changes in proteinuria evidence, making it a handy tool for monitoring disease activity and treatment response [8]. While more research should be performed to understand better the utility of trending these levels, it can prove helpful to monitor treatment efficacy and progression more so than proteinuria, which may be less specific to disease activity.

## Conclusions

While membranous nephropathy may be less common, it is imperative to consider this differential when suspicious of nephrotic syndrome. Furthermore, the increasing recognition of MN as an autoimmune-mediated disease highlights the importance of considering it despite encountering a less classic initial presentation and patient demographic, as in the case of our patient. The importance of obtaining imaging and a thorough workup is also crucial to avoid delaying treatment and diagnosis of the disease and its complications.

## References

[1] Keri KC, Blumenthal S, Kulkarni V, Beck L, Chongkrairatanakul T (2019). Primary membranous nephropathy: comprehensive review and historical perspective. Postgrad Med J.

[2] Mathieson PW (2012). Membranous nephropathy. Clin Med (Lond).

[3] Asghar M, Ahmed K, Shah SS, Siddique MK, Dasgupta P, Khan MS (2007). Renal vein thrombosis. Eur J Vasc Endovasc Surg.

[4] Zhu H, Xu L, Liu X, Liu B, Zhai C, Wang R, Yang X (2022). Anti-PLA2R antibody measured by ELISA predicts the risk of vein thrombosis in patients with primary membranous nephropathy. Ren Fail.

[5] Beck LH Jr, Bonegio RG, Lambeau G (2009). M-type phospholipase A2 receptor as target antigen in idiopathic membranous nephropathy. N Engl J Med.

[6] Pham PC, Pham PT (2021). Pham P-C, T. Pham P-T: Nephrology and Hypertension Board Review. Lippincott Williams & Wilkins, Philadelphia, PA; 2021. Nephrology and hypertension board review.

[7] Kanigicherla D, Gummadova J, McKenzie EA (2013). Anti-PLA2R antibodies measured by ELISA predict long-term outcome in a prevalent population of patients with idiopathic membranous nephropathy. Kidney Int.

[8] Segal PE, Choi MJ (2012). Recent advances and prognosis in idiopathic membranous nephropathy. Adv Chronic Kidney Dis.

